# Bad Breath

**Published:** 1871-02

**Authors:** 


Bad Breath, Cause and Remedy.—A bad breath is an indication of imperfect digestion of the food, and whatever tends to derange the digestive organs, is a producing cause of a disagreeable breath. Among these causes are eating at irregular hours, too often and too fast, eating too large a quantity and an undue proportion of carbonaceous food, as fat meats, grease, butter, sugar, candies, cakes, pastry, starch, fine flour, etc., the use of tobacco and alcoholic liquors, inattention to cleanliness, and lack of sufficient exercise. A bad breath is sometimes caused by decayed teeth, but these in turn are usually intimately affected by the condition of the stomach. The teeth must be kept scrupulously clean, and where decay has taken place they should be filled with gold, if they can be saved, if not, extracted at once.—*Herald of Health.*

